# High-zinc diets accelerate molting and recovery by remodeling the cecal microbiome in laying hens

**DOI:** 10.3389/fmicb.2026.1743943

**Published:** 2026-05-13

**Authors:** Ling Chang, Fang Peng, Yue Zuo, Shihao Yang, Kun Xie, Zehe Song, Xi He, Yuguang Chen

**Affiliations:** 1 College of Animal Science and Technology, Hunan Agricultural University, Changsha, Hunan, China; 2 Hunan Engineering Research Center of Poultry Production Safety, Changsha, Hunan, China; 3 Yuelushan Laboratory, Hunan Agricultural University, Changsha, Hunan, China; 4 Beijing No.80 High School, Beijing, China

**Keywords:** amino acid metabolism, gut microbiota, gut-ovary axis, high-zinc, molting

## Abstract

**Introduction:**

Conventional fasting molting can restore laying performance but imposes substantial physiological stress and welfare concerns. High-zinc diets (2% ZnO) have been proposed as a less stressful alternative, but their mechanisms remain unclear.

**Methods:**

This study compared fasting-induced molting with zinc oxide supplementation in 384 Lohmann Pink hens (65 wk). Serum, cecal contents, and ovaries were sampled across six stages from pre-molt to post-molt recovery.

**Results:**

ZnO accelerated cessation of lay (5.75 vs. 8.87 d) and earlier recovery to 50% production (15.25 vs. 16.62 d) with lower weight loss (25% vs. 30%). Multi-omics revealed that high-zinc feeding enriched beneficial microbes (e.g., *Coprenecus pullicola*, *Fournierella* spp.) involved in amino acid and cofactor biosynthesis, consistent with activated glycine/serine/lysine metabolism. ZnO also reduced inflammatory and barrier-injury signals (IL-1β, LPS, DAO) and promoted earlier recovery of reproductive hormones (higher IGF-1, earlier increases in E2 and LH). Both treatments improved post-molt egg quality; zinc better preserved albumen height and Haugh unit, while fasting increased shell thickness and yolk color.

**Discussion/conclusion:**

These findings support that a high-zinc-associated microbial-metabolic profile mitigates stress, accelerates ovarian reset, and shortens the time to restore production.

## Graphical Abstract

**Figure:**
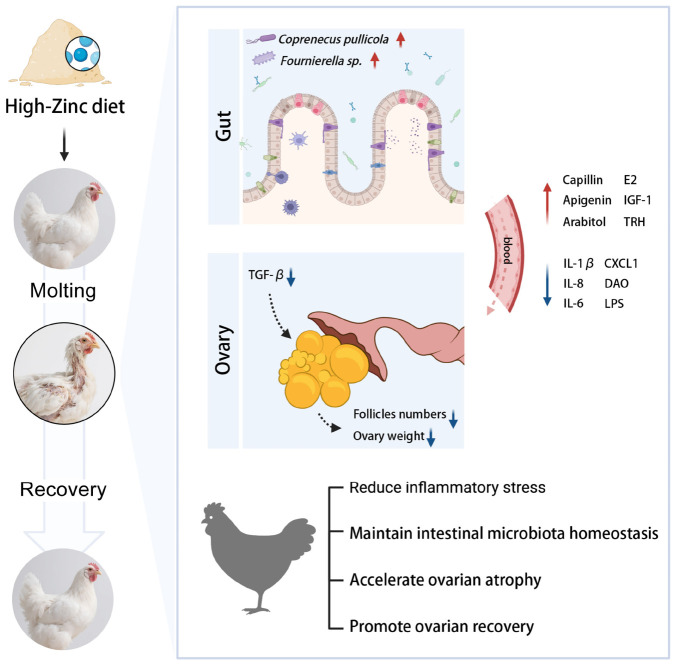
High-zinc diet (2% ZnO) accelerates molting and recovery in laying hens by remodeling cecal microbiota, reducing inflammatory stress, and promoting ovarian regeneration via the gut–ovary axis. It was created with BioRender.com under a publication license.

## 1 Introduction

The laying performance of commercial hens declines with age, leading to reduced productivity and economic efficiency in the poultry industry (Andreatti Filho et al., 2019). Artificial forced molting, a process that temporarily halts egg production to promote feather regeneration, is commonly used to extend hens’ productive lifespan and restore laying performance (Mishra et al., 2022; Sun et al., 2025). Traditional molting methods typically rely on feed restriction, often combined with water withdrawal or light manipulation. Although these methods are simple, they can cause significant physiological stress, including rapid body weight loss (25–30%), impaired intestinal barrier integrity, and systemic inflammation, which can increase mortality rates (10–15%) during molting and raise serious animal welfare concerns (Berry, 2003).

To address these welfare issues, high-zinc diets (10,000–20,000 ppm Zn) have emerged as a more humane alternative to conventional molting methods. Zinc supplementation effectively suppresses egg production within 5–6 days, strengthens eggshells, and reduces breakage rates (Shippee et al., 1979). Compared to fasting-induced molting, zinc supplementation has been shown to induce less physiological stress (Khan et al., 2011), improve operational efficiency, and enhance post-molt egg quality. Nevertheless, the use of very high dietary Zn also raises practical considerations, including regulatory limits on trace mineral inclusion and potential environmental concerns related to increased Zn excretion and manure management, which may influence the feasibility of field implementation. Despite these well-documented production and welfare benefits, the underlying mechanisms, particularly the role of gut microbiota in zinc-induced molting, remain poorly understood.

The gut microbiota serves as a crucial regulator of host metabolism and immunity, significantly influencing reproductive physiology through the gut-ovary axis (Xu et al., 2023; Zhu, 2023). Zinc plays a pivotal role in shaping microbial composition, with high-zinc diets promoting the proliferation of short-chain fatty acid (SCFA)-producing genera, including *Faecalibacterium* and members of *Clostridiales* (Eeckhaut et al., 2011; Rychlik, 2020; Skalny et al., 2021). These metabolites, particularly butyrate, inhibit histone deacetylases and modulate ovarian gene expression (Scarpellini et al., 2022). Furthermore, zinc contributes to the maintenance of intestinal barrier integrity, thereby reducing endotoxin translocation and alleviating reproductive inflammation (Nasiadek et al., 2020). Despite these findings, the exact mechanisms through which zinc-induced alterations in microbiota and their metabolic products influence molting efficiency remain poorly understood.

We hypothesize that the high-zinc molting regimen may improve molting efficiency, in part through alterations in gut microbiota composition and metabolic activity, thereby enhancing host metabolism and promoting ovarian recovery. Molting efficiency was quantified by body weight change (pre-induction to molt completion) and production milestones (days to cessation and resumption of lay, 50% egg production, and peak lay). To elucidate the biological basis of these phenotypes, we integrated metagenomic sequencing and untargeted metabolomics with assessments of egg quality, follicular development, serum reproductive hormones, and inflammation-related factors. This study aims to provide new insights into the role of gut microbiota in the molting process of aged laying hens, with a focus on gut–ovary interactions and metabolic recovery.

## 2 Materials and methods

### 2.1 Ethics statement

All procedures were approved by the Animal Ethics Committee of Hunan Agricultural University (Protocol No. HAU ACC 2023DKJQ097).

### 2.2 Experimental design

A total of 384 Lohmann Pink laying hens from the same flock were used in this study. All birds were reared under consistent routine feeding and management conditions from 40 to 65 weeks of age, without any additional experimental intervention before the formal molting trial. At 40 weeks of age, the laying rate was approximately 95%, and by 65 weeks of age it had declined naturally to approximately 75%. At 65 weeks of age, the hens were randomly assigned to one of two molting induction treatments: a fasting group (Fast) and a high-zinc diet group (Zn). Each treatment included 16 replicate pens, with 12 hens per pen (*n* = 192 hens per treatment). The experimental environment was controlled, maintaining an ambient temperature between 13 and 25°C and a relative humidity of 60–70%.

### 2.3 Molting induction procedures

In the Fast group, molting was induced through feed withdrawal. During the initial 3 days, hens received a limited quantity of crushed corn and grit. From day four onward, feed was completely withdrawn, and water was withheld on days eight and nine. The photoperiod was reduced to 8 h of light per day at a luminosity of ≤ 5 lux.

In the Zn group, hens had continuous access to water and were fed a basal diet formulated in accordance with the Chinese National Standard GB/T 5916–2020 (Table 1), supplemented with 2% zinc oxide (ZnO). The photoperiod in this group was maintained at 12 h of light per day at a luminosity of ≥ 50 lux.

**TABLE 1:** Ingredient composition and nutrient level of the basal diets (dry basis) %.

Ingredients	Content, %
Corn	40.07
Soybean meal	20.30
Rough rice	20.00
Limestone	9.00
Wheat bran	2.20
Corn gluten meal (CGM)	2.00
Rapeseed meal	2.00
Dicalcium phosphate (CaHPO4)	1.33
Soybean oil	0.60
Premix^1^	2.50
Total	100.00
Nutrient levels^2^	
ME, MJ/kg	10.80
Crude protein	16.84
Ether extract	2.96
Crude fiber	2.43
Calcium	3.95
Available Ca	3.56
Total phosphorus	0.66
Available P	0.33
Methionine	0.40
Lysine	0.79

^1^The premix provided the following (per kilogram of diet): vitamin A, 10,000 IU; vitamin D3, 2,500 IU; vitamin E, 27.0 IU; vitamin K3, 3.0 mg; vitamin B1, 1.0 mg; vitamin B2, 4 mg; nicotinic acid, 32 mg; pantothenic acid, 11 mg; vitamin B6, 3.0 mg; folic acid, 0.5 mg; vitamin B12, 25 mg; biotin, 50.0 mg; Fe, 60.0 mg; Zn, 60.0 mg; Mn, 100.0 mg; Cu, 5.0 mg; I, 0.5 mg; Se, 0.2 mg.

^2^The nutrient levels were calculated values.

**FIGURE 1:**
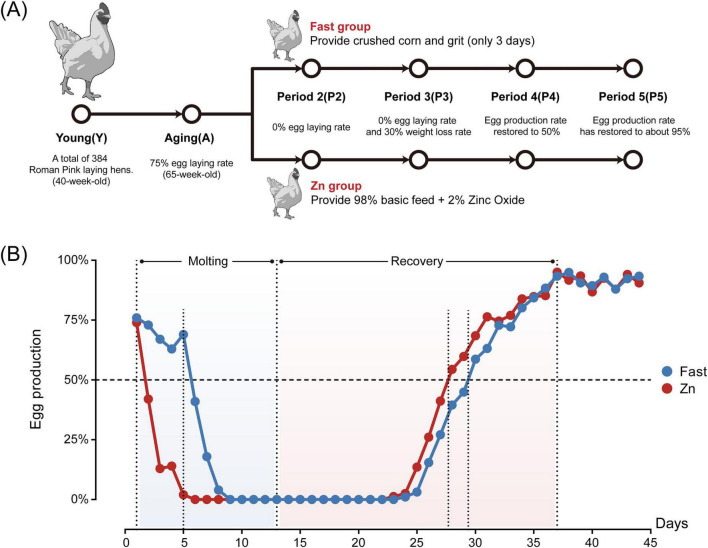
Experimental design and egg production curve. **(A)** Experimental timeline and sampling scheme. “Fast group”: crushed corn and grit for 3 d, “Zn group”: 98% basal diet + 2% ZnO. Sampling/staging: Y (young, 40-wk), A (pre-molt, 65-wk), P2 (cessation of lay), P3 (molting completion; ∼30% body-weight loss in Fast), P4 (50% production recovery), and P5 (peak lay restoration). **(B)** Daily egg-production curves (%) from induction through recovery for both groups. Shaded regions mark the Molting and Recovery phases; vertical dotted lines indicate stage boundaries (P2–P5), and the dashed horizontal line marks 50% production. Points denote daily means.

Body weight was monitored every 3–5 days, with one replicate cage randomly selected from each treatment group. The percentage of body weight loss was calculated relative to the pre-molt baseline. Molting induction in the Fast group was deemed complete once the average body weight loss reached 30%. The molting phase for the Zn group concluded simultaneously. Following molting, both groups were transitioned to a standard basal diet under normal feeding and lighting conditions to facilitate post-molt recovery. A schematic overview of the experimental design and sampling timeline is presented in Figure 1A.

### 2.4 Laying rate and sample collection

Eggs were collected daily at 9:00 a.m. to monitor egg production. The daily laying rate was calculated for each replicate pen as follows: laying rate (%) = (number of eggs produced per pen per day/number of live hens present in that pen per day) × 100. Pen-level laying rate data were then used to determine the main production milestones, including days to cessation of lay, days to resumption of lay, days to 50% egg production, and days to peak lay.

Biological sampling was conducted at six key physiological stages: young (**Y**, 40 weeks of age), pre-molt (**A**, 1 day before molting at 65 weeks), cessation of lay (**P2**), molting completion (**P3**, corresponding to a 30% body weight loss in the Fast group), 50% production recovery (**P4**), and peak lay restoration (**P5**). At each sampling stage, eight individual hens were randomly selected from each treatment group. Briefly, eight of the 16 replicate pens were randomly selected, and one hen per selected pen was sampled. At subsequent stages, pens were re-randomized with preference for those not sampled at the previous stage, ensuring that hens were independently sampled at each stage. Blood samples were obtained from the wing vein using 5 mL vacutainer tubes (no anticoagulant). Following clot formation, samples were centrifuged at 1,500 × g for 15 min at 4°C to separate the serum. Serum was aliquoted into pre-labeled sterile microtubes and stored at -80°C until analysis. Serum was used for hormone profiling, inflammatory-marker quantification, and untargeted metabolomics; hemolyzed samples were excluded.

Hens were euthanized by CO_2_ inhalation in accordance with the AVMA Guidelines for the Euthanasia of Animals (Leary, 2020) and the Guide for the Care and Use of Agricultural Animals in Research and Teaching (Vaughn, 2012). CO_2_ (99.9% purity) was delivered from a compressed-gas cylinder into a sealed chamber, and hens were monitored continuously until death was confirmed. Immediately thereafter, ovaries were aseptically excised, rinsed in ice-cold PBS, blotted dry, and follicles were counted and classified by size (white, yellow, and hierarchical). Cecal chyme was collected, homogenized, aliquoted into sterile microtubes, and stored at -80°C for metagenomic analysis.

### 2.5 Egg quality

Egg quality was assessed at three distinct stages: young hens (**Y**, 40 weeks), pre-molt aged hens (**A**, 65 weeks), and post-molt peak production (**P5**). For **Y** and **A**, eggs were obtained as stage-level random samples without tracking pen identity: 80 eggs were randomly collected for baseline egg quality assessment. For **P5** (peak lay restoration), egg quality was evaluated at the pen level. Specifically, five eggs per pen were randomly collected from each replicate pen in the Fast and Zn groups (16 pens per treatment; 80 eggs per treatment).

Egg quality parameters, including egg weight, albumen height, yolk color, and Haugh unit, were measured using an ORKA EA-01 Egg Quality Analyzer (ORKA Food Technology Ltd., Herzliya, Israel). Eggshell strength was determined using an EFR-01 Eggshell Strength Tester (ORKA Food Technology Ltd., Herzliya, Israel). The thickness of eggshell (excluding the inner membrane) was measured at three positions: the blunt end, sharp end, and equatorial region using a digital vernier caliper (Model ARZ-1331, AIRAJ, China).

### 2.6 Follicles count

Follicles were categorized by diameter into three groups: white follicles (1-6 mm), yellow follicles (6-8 mm), and pre-ovulatory follicles (> 9 mm). The diameter of each follicle was measured using a caliper, and the follicles were counted according to their size.

### 2.7 Measurement of serum endocrine hormones and inflammation-related factors

Serum concentrations of estradiol (E2; MB-4608A), luteinizing hormone (LH; MB-2659A), insulin-like growth factor 1 (IGF-1; MB-2650A), transforming growth factor β (TGF-β; MB-19693A), thyrotropin-releasing hormone (TRH; MB-9430A), cortisol (CORT; MB-19619A), interleukin-1β (IL-1β; MB-5248A), interleukin-6 (IL-6; MB-5250A), and interleukin-8 (IL-8; MB-9558A) were measured using chicken-specific enzyme-linked immunosorbent assay (ELISA) kits following the manufacturers’ protocols (Jiangsu Meibiao Biotechnology Co., Ltd., Nanjing, China). Lipopolysaccharides (LPS; ml059937), chemokine (C–X–C motif) ligand 1 (CXCL1; ml037006), and diamine oxidase (DAO; ml036981) were measured using ELISA kits from Shanghai Enzyme-linked Biotechnology Co., Ltd. (Shanghai, China).

### 2.8 Microbial DNA extraction and metagenomics analysis

The following steps were conducted by Shanghai Personal Biotechnology Co., Ltd. (Shanghai, China). Total microbial genomic DNA from cecal content (*n* = 7 per treatment per stage; A, P2, P3, P4, and P5) was extracted using an OMEGA Soil DNA kit (D5625-01, OMEGA, United States). The quantity and quality of extracted DNA were measured using a NanoDrop ND-1000 spectrophotometer (Thermo Fisher Scientific, Waltham, MA, United States) and agarose gel electrophoresis, respectively. The extracted microbial DNA was processed to construct metagenome shotgun sequencing libraries with insert sizes of 400 bp by using Illumina TruSeq Nano DNA LT Library Preparation kit (Illumina, United States). Each library was sequenced by Illumina HiSeq X-ten platform (Illumina, United States) with PE150 strategy at Personal Biotechnology Co., Ltd. (Shanghai, China).

Raw sequencing reads were processed to obtain quality-filtered reads for further analysis. First, sequencing adapters were removed from sequencing reads using Cutadapt (v.1.2.1) (Kechin et al., 2017). Secondly, low quality reads were trimmed using a sliding-window algorithm in fastp (Chen et al., 2018). Thirdly, reads were aligned to the host genome of broiler using BMTagger to remove host contamination. Once quality-filtered reads were obtained, taxonomical classifications of metagenomics sequencing reads from each sample were performed using Kraken2 (v2.1.3) (Wood et al., 2019) against an RefSeq-derived database, which included genomes from archaea, bacteria, viruses, fungi, protozoans, metazoans and Viridiplantae. CDS sequences of all samples were clustered by mmseqs2 (Steinegger and Söding, 2017) with “easy-cluster” mode, setting protein sequence identity threshold to 0.90 and covered residue of the shorter contig to 90%. The high-quality reads of each sample were aligned against the gene catalog by Salmon (Patro et al., 2017) to calculate relative gene abundance.

Beta diversity analysis was performed to investigate the compositional and functional variation of microbial communities across samples using Bray–Curtis distance metrics and visualized via principal coordinate analysis (PCoA). Based on the taxonomic and functional profiles of non-redundant genes, linear discriminant analysis effect size (LEfSe) was performed to detect differentially abundant taxa and functions across groups using the default settings (Kruskal–Wallis test, *P* < 0.05; LDA score > 2). The functionality of the non-redundant genes was obtained by annotation using MMseqs2 in “search” mode against the KEGG, EggNOG, and CAZy protein databases. Differences in the abundance of each functional unit between sample groups were assessed using the default statistical test implemented in the pipeline (Student’s *t*-test and/or Kruskal–Wallis test, as appropriate). *P-*values were adjusted for multiple testing using the Benjamini–Hochberg false discovery rate (FDR) procedure, and features with FDR-adjusted *P* (adj. *P*) < 0.05 were considered statistically significant and used for downstream visualization.

### 2.9 Serum metabolomic analysis

The following steps were conducted by Shanghai Personal Biotechnology Co., Ltd. (Shanghai, China). Fifty microliters (50 μL) of serum (*n* = 8 per treatment per stage; A, P2, P3, P4, and P5) were mixed with 300 μL of pure methanol, vortexed for 10 s, and then centrifuged at 12,000 r/min for 10 min at 4°C. The supernatant was collected and centrifuged again at 12,000 r/min for an additional 5 min at 4°C. Following refrigeration at -20°C for 30 min, the sample was centrifuged again at 12,000 r/min at 4°C for 3 min, and 150 μL of the supernatant was transferred to an injection vial for analysis. The sample extracts were then analyzed using a liquid chromatography-electrospray ionization-tandem mass spectrometry (LC-ESI-MS/MS) system, equipped with a Waters ACQUITY UPLC HSS T3 C18 column, under specific analytical conditions. The mass spectrometry analysis was performed in both positive and negative ion modes, with multiple reaction monitoring transitions monitored for each period based on the metabolites eluted within that period.

### 2.10 Statistical analyses

All statistical analyses were performed in SPSS 27.0 (IBM, Chicago, IL, United States) and are reported as mean ± standard deviation (SD). Depending on the experimental unit and data structure, outcomes were analyzed using the General Linear Model (GLM), two-way GLM/ANOVA (treatment, stage, and their interaction), and ANCOVA. Where applicable, stage-specific contrasts were evaluated using estimated marginal means with multiple-comparison adjustment (e.g., Bonferroni). For selected pairwise comparisons without equal-variance assumptions, Welch’s two-tailed *t*-test was used. For multi-omics feature-level analyses, *P-*values were adjusted using the Benjamini–Hochberg false discovery rate (FDR) procedure, with *q* < 0.05 considered significant. Unless otherwise stated, two-sided *P* < 0.05 was considered statistically significant. Figures were prepared using GraphPad Prism 9 (San Diego, CA, United States).

## 3 Results

### 3.1 High-zinc diet accelerates molt onset and post-molt production recovery compared to fasting

Before molting, no significant differences in body weight were observed between the Fast and Zn groups (*P* > 0.05); however, by the end of molting, the body weight of the Fast group was significantly lower than that of the Zn group (*P* = 0.036). Following the initiation of molting, both groups exhibited a rapid decline in egg production (Figure 1B). Notably, the Zn group ceased egg production significantly earlier (5.75 ± 0.45 days) compared to the Fast group (8.87 ± 0.34 days) (*P* < 0.001) (Table 2). Upon completion of molting, defined as a 25-30% body weight loss in the Fast group, the Zn group tended to exhibit a lower body weight loss (25% ± 0.03%) relative to the Fast group (30% ± 0.06%) (*P* = 0.055).

**TABLE 2:** Comparison of body weight and key egg production milestones in hens induced by fast and high-zinc diets.^1^

Items	Fast	Zn	*P*-value
Body weight before induction (kg/hen)	1.87 ± 0.08	1.90 ± 0.17	0.674
Body weight at molting completion (kg/hen)^2^	1.30 ± 0.12	1.47 ± 0.19	0.036
Body weight loss	0.30 ± 0.06	0.25 ± 0.03	0.055
Days to cessation of lay (d)^3^	8.87 ± 0.34	5.75 ± 0.45	<0.001
Days to resumption of lay (d)	12.62 ± 0.88	11.87 ± 0.96	0.029
Days to 50% egg production (d)	16.62 ± 1.78	15.25 ± 1.44	0.023
Days to peak lay (d)^4^	21.25 ± 3.30	20.06 ± 4.05	0.371

^1^Data represent means from 16 replicates (i.e., pens) per treatment. *P-*values were obtained using GLM with treatment as a fixed effect.

^2^Body weight at molting completion was analyzed by ANCOVA with pre-induction body weight as a covariate.

^3^All days are counted from the start of molting induction (Day 0).

^4^Peak lay is defined as the maximum weekly laying rate computed with a 7-day rolling window.

During the recovery phase, the Zn group resumed egg production significantly earlier (11.87 ± 0.96 days) than the Fast group (12.62 ± 0.88 days) (*P* < 0.05) and achieved 50% egg production at a faster rate (15.25 ± 1.44 days vs. 16.62 ± 1.78 days) (*P* < 0.05) (Table 2). However, the time taken to reach peak egg production did not differ significantly between the two groups.

### 3.2 Molting improves egg quality parameters with diet-specific effects

**FIGURE 2:**
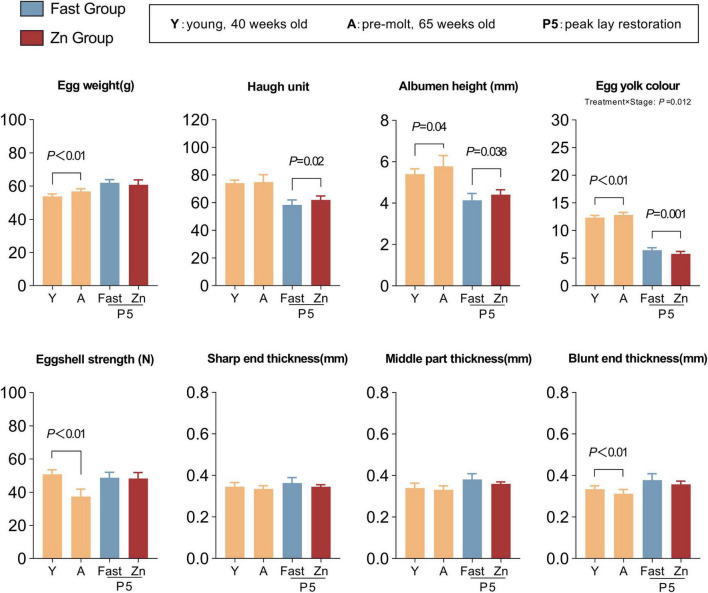
Egg-quality traits across age and post-molt recovery. Bar plots show egg weight, Haugh unit, albumen height, yolk color, eggshell strength, and eggshell thickness at the sharp, middle, and blunt ends. Categories are Y (young, 40 wk), A (pre-molt, 65 wk), and P5 (peak lay restoration; Fast vs. Zn). Error bars indicate SD. Y vs. A comparisons were conducted at the egg level (random eggs without pen tracking) using Welch’s two-tailed *t*-test/GLM and are presented as stage-level reference. At P5, treatment effects were evaluated on pen means (five eggs per pen; *n* = 16 pens per treatment) using Welch’s two-tailed *t*-test (Fast vs. Zn). Brackets indicate the corresponding pairwise comparisons with *P-*values.

Egg quality was evaluated at three distinct stages: young (Y, 40 weeks), pre-molt (A, 65 weeks), and post-molt peak production (P5). As hens aged from the Y stage to the A stage, egg weight, albumen height, and yolk color increased significantly (*P* < 0.05), whereas eggshell strength and blunt end thickness showed marked decreases (*P* < 0.001) (Figure 2). Notably, pronounced treatment-specific effects emerged during the P5 stage. Specifically, compared with the Fast group, the Zn group exhibited higher Haugh units and albumen height (*P* < 0.05), while yolk color was significantly reduced (*P* < 0.01) (Figure 2).

### 3.3 Follicular dynamics and ovarian weight changes during molting and recovery

**FIGURE 3:**
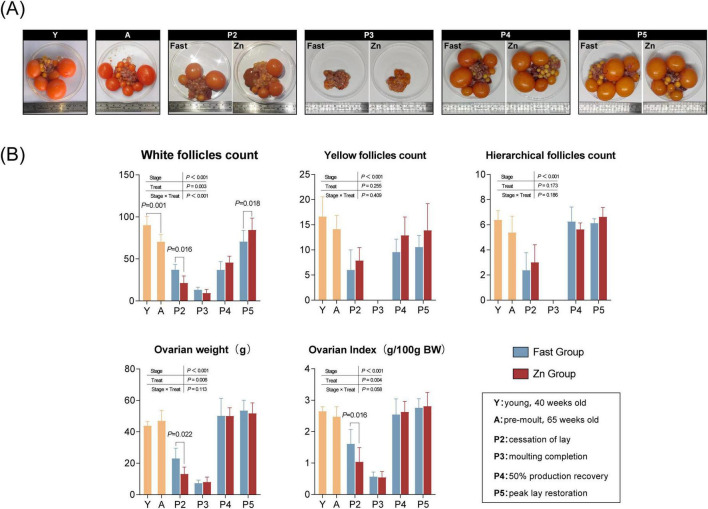
Ovarian morphology and follicle/ovary traits across stages. **(A)** Representative ovaries at Y (young, 40 wk), A (pre-molt, 65 wk), P2 (cessation of lay), P3 (molting completion), P4 (50% production recovery), and P5 (peak lay restoration), illustrating regression and recovery over the molting cycle. **(B)** Quantification of white follicles (1–6 mm), yellow follicles (6–8 mm), hierarchical follicles (≥ 9 mm), ovarian weight, and ovarian index (g/100 g body weight). Bars show mean ± SD (*n* = 8 per stage). Y vs. A differences were evaluated using Welch’s two-tailed *t*-test/GLM as stage-level reference. Treatment effects during molting were analyzed using P2–P5 data in a two-way GLM/ANOVA with treatment (Fast vs. Zn), stage (P2–P5), and their interaction; A values are shown as descriptive baseline reference. Brackets indicate stage-specific Fast vs. Zn contrasts with the corresponding *P-*values.

Ovarian follicle counts and ovarian weights exhibited dynamic changes throughout the study, and the macroscopic morphology of ovaries at different stages is presented in Figure 3A; broadly similar patterns were observed between the Fast and Zn groups; however, significant differences emerged at specific time points. At 40 weeks of age (Y), hens exhibited the highest numbers of white follicles, yellow follicles, and hierarchical follicles. By the pre-molt stage (A, 65 weeks), the number of white follicles had significantly declined (*P* < 0.001) (Figure 3B).

During the post-induction period (P2–P5), ovarian traits exhibited pronounced stage-dependent dynamics. Two-way analysis indicated that the number of white follicles (1–6 mm) varied significantly across stages and exhibited a treatment × stage interaction, with between-treatment differences emerging at specific time points rather than uniformly throughout the recovery period. Specifically, at stage P2, the Zn group showed a significantly lower number of white follicles compared with the Fast group (*P* < 0.05). In contrast, yellow follicles (6–8 mm) and hierarchical follicles (≥ 9 mm) were primarily influenced by stage effects (*P* < 0.05), and no consistent treatment-related differences were observed across P2–P5. Similarly, ovarian weight and ovarian index changed markedly over time, with significant stage effects detected (*P* < 0.05). Although overall treatment effects were observed for both ovarian weight and ovarian index (*P* < 0.05), the magnitude of these differences was stage-dependent and most pronounced during the early post-induction stage (P2) whereas contrasts at later stages showed less consistent separation between the Fast and Zn groups.

### 3.4 Hormonal dynamics during induced molting reveal diet-specific restoration patterns

**FIGURE 4:**
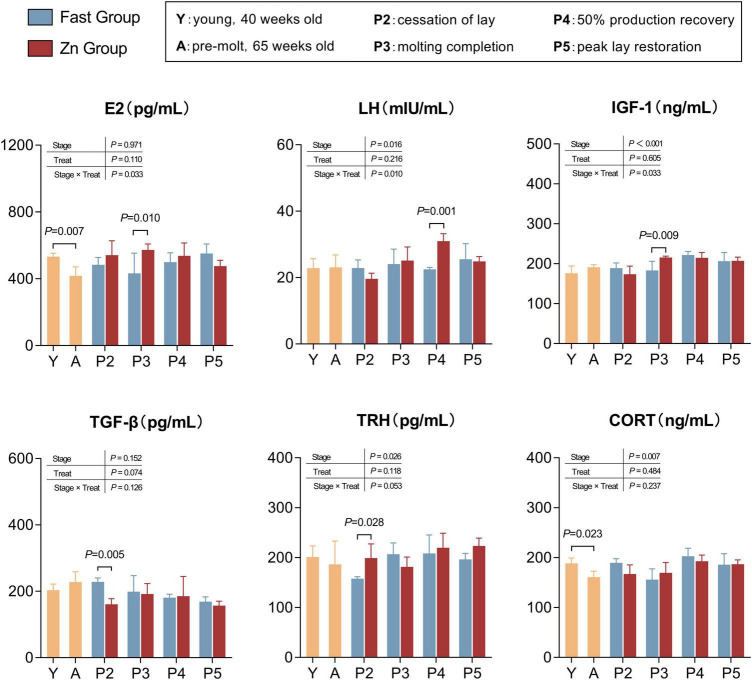
Endocrine profiles across induction and recovery stages. Serum concentrations of estradiol (E2), luteinizing hormone (LH), insulin-like growth factor-1 (IGF-1), transforming growth factor-β (TGF-β), thyrotropin-releasing hormone (TRH), and corticosterone (CORT) measured at Y, A, and P2–P5. Bars show mean ± SD (*n* = 8 per stage). Y vs. A differences were assessed using Welch’s two-tailed *t*-test/GLM as stage-level reference. Treatment effects during molting were evaluated using P2–P5 data in a two-way GLM/ANOVA with treatment (Fast vs. Zn), stage (P2–P5), and their interaction; A values are presented descriptively as baseline reference for treatment comparisons. Brackets indicate stage-specific Fast vs. Zn contrasts with corresponding *P-*values.

As shown in Figure 4. Circulating estradiol (E2) and corticosterone (CORT) levels declined significantly from the young stage (Y) to the pre-molt stage (A) (*P* < 0.05). During the post-induction period (P2–P5), two-way analysis incorporating treatment, stage, and their interaction revealed pronounced stage-dependent endocrine responses. Specifically, E2 levels varied significantly across stages and exhibited a treatment × stage interaction. The Zn group displayed higher E2 levels during the early recovery phase, particularly around stages P3–P4, but lower E2 levels by stage P5 compared with the Fast group (*P* < 0.05 or trend).

Similarly, luteinizing hormone (LH) showed a stage-dependent treatment effect, with significantly higher levels observed in the Zn group at stage P4 (*P* < 0.05). In parallel, insulin-like growth factor 1 (IGF-1) changed markedly over time, reaching peak levels around stage P4 (stage effect, *P* < 0.05), which is consistent with enhanced metabolic recovery during this period.

With respect to transforming growth factor-β (TGF-β), the Zn group exhibited a pronounced reduction at stage P2 relative to the Fast group (*P* < 0.05), whereas differences between treatments became less evident at later stages. In addition, thyrotropin-releasing hormone (TRH) levels were higher in the Zn group at stage P5 (*P* < 0.05). By contrast, CORT levels increased progressively across recovery stages (stage effect, *P* < 0.05) but did not show consistent between-treatment differences at individual time points.

### 3.5 Differential inflammatory responses during molting reveal gut-immune axis modulation

**FIGURE 5:**
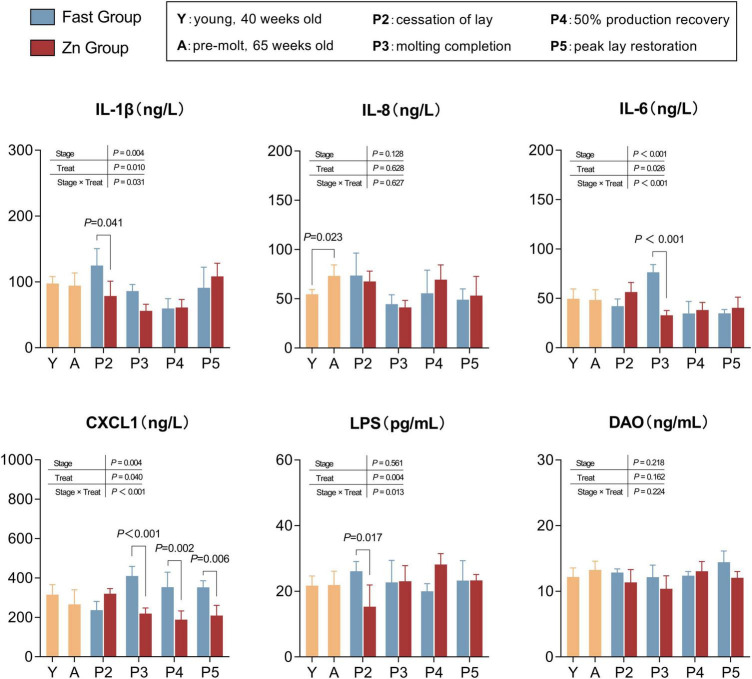
Inflammation and epithelial-injury markers across stages. Serum IL-1β, IL-8, IL-6, CXCL1, LPS, and DAO measured at Y, A, and P2–P5. Bars show mean ± SD (*n* = 8 per stage). Y vs. A differences were assessed using Welch’s two-tailed *t*-test/GLM as stage-level reference. Treatment effects during molting were evaluated using P2–P5 data in a two-way GLM/ANOVA with treatment (Fast vs. Zn), stage (P2–P5), and their interaction; A values are presented descriptively as baseline reference for treatment comparisons. Brackets indicate stage-specific Fast vs. Zn contrasts with corresponding *P-*values.

As shown in Figure 5. Inflammatory and epithelial injury–related markers exhibited dynamic changes during the post-induction period (P2–P5), with several endpoints demonstrating stage-dependent treatment effects. Two-way analysis revealed significant treatment × stage interactions for IL-1β (*P* = 0.031), IL-6 (*P* < 0.001), CXCL1 (*P* < 0.001), and LPS (*P* = 0.013), indicating that both the magnitude and direction of Zn-associated effects varied across recovery stages.

Stage-specific contrasts with multiple-testing adjustment further demonstrated that IL-1β levels were significantly lower in the Zn group at stage P2 (*P* = 0.041), whereas no differences were detected at later stages. Similarly, IL-6 was markedly reduced by Zn at stage P3 (*P* < 0.001). In addition, CXCL1 levels were consistently lower in the Zn group from stages P3 through P5 (*P* ≤ 0.006), suggesting a sustained modulatory effect during the later recovery phase.

LPS levels were also reduced in the Zn group at stage P2 (*P* = 0.017); however, differences at subsequent stages were not statistically significant. In contrast, IL-8 and diamine oxidase (DAO) did not exhibit significant main effects or treatment × stage interactions, and no stage-specific differences remained significant after adjustment (*P* > 0.05).

### 3.6 High-zinc diet and fasting induce period-specific shifts in gut microbiota structure

**FIGURE 6:**
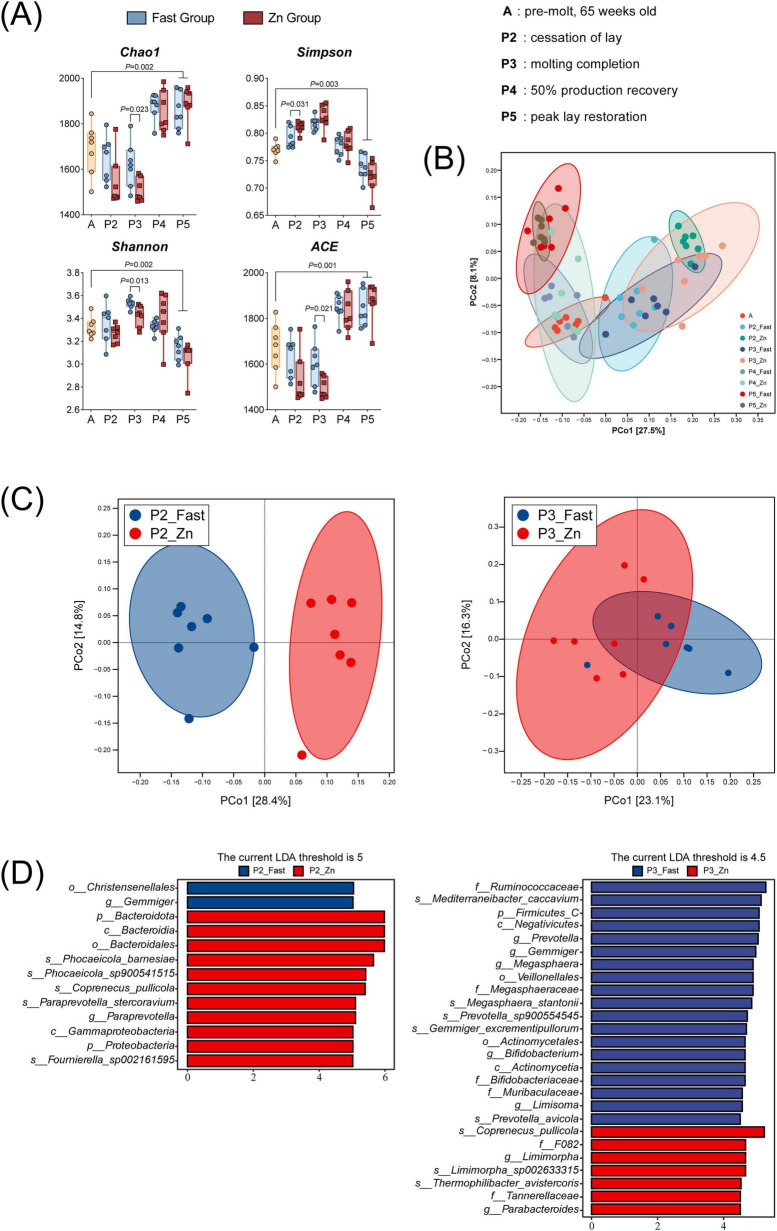
Microbiome diversity and taxa distinguishing fasting vs. zinc feeding. **(A)** Alpha-diversity indices (Chao1, Shannon, Simpson, ACE) at A and P2-P5 for Fast and Zn groups (*n* = 7 per stage). **(B)** Principal coordinates analysis (PCoA, Bray-Curtis distances) of community structure across stages (*n* = 7 per stage). **(C)** Stage-specific PCoA for P2 and P3 highlighting group separation (*n* = 7 per stage). **(D)** LEfSe results identifying taxa discriminating groups at P2 (left; LDA threshold 5.0) and P3 (right; LDA threshold 4.5). Bars indicate LDA scores for taxa enriched in Fast (blue) or Zn (red); taxonomic ranks are labeled at phylum and species. Metagenomic analyses were performed using *n* = 7 biological replicates per treatment per stage.

Significant shifts in cecal microbiota composition occurred during molting, with marked differences between the Zn and Fast groups at key stages (P2 and P3). At P5, post-molt microbiota showed significantly increased species richness (Chao1 and ACE indices; *P* < 0.05), but decreased microbial diversity (Shannon index; *P* < 0.05) and evenness (Simpson index; *P* < 0.05) compared to pre-molt (A) (Figure 6A; Supplementary Table 1). PCoA based on Bray-Curtis distances revealed significant shifts in β-diversity at P5, with clear separation between pre-molt (A) and post-molt (P5) microbiota (Figure 6B).

During molting induction (P2-P3), α-diversity metrics differed between treatments at specific time points. At P2, the Zn group showed a higher Simpson index than the Fast group (*P* < 0.05), indicating reduced community evenness. However, at P3, the Fast group exhibited greater richness (Chao1, ACE) and diversity (Shannon) compared to the Zn group (*P* < 0.05) (Figure 6C).

β-diversity analysis further emphasized period-specific structural differences, with significant separation between the Zn and Fast groups at P2 (Figure 6C). The Zn group was enriched with differentially abundant taxa at P2, such as *Phocaeicola barnesiae*, *Coprenecus pullicola*, *Paraprevotella stercoravium*, and *Fournierella sp002161595* (Figure 6D). At P3, *Coprenecus pullicola* remained the predominant taxon in the Zn group, indicating a distinct microbial composition compared to the Fast group.

### 3.7 Dietary zinc and fasting induce divergent functional profiles in gut microbiota during molting induction

**FIGURE 7:**
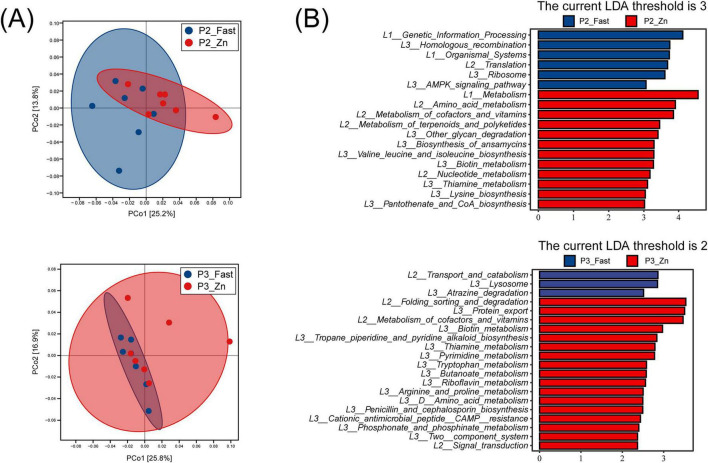
Functional metagenomic differences between fasting and zinc feeding. **(A)** PCoA based on KEGG functional profiles (gene families collapsed to KEGG level 3) derived from metagenomic data (*n* = 7 per stage). **(B)** LEfSe of KEGG functions showing pathways enriched in Fast (blue) vs. Zn (red) at P2 (top; LDA threshold 3.0) and P3 (bottom; LDA threshold 2.0). Axes show LDA scores. Metagenomic analyses were performed using *n* = 7 biological replicates per treatment per stage.

KEGG pathway analysis revealed significant functional differences in the cecal microbiome between the Zn and Fast groups during molting induction (P2 and P3) (Figure 7A). At P2, the Zn group exhibited significant enrichment in pathways related to nutrient metabolism, including amino acid metabolism, cofactor and vitamin biosynthesis, and nucleotide metabolism. Specific pathways such as biotin metabolism, thiamine metabolism, and terpenoid/polyketide metabolism were notably enriched (Figure 7B). In contrast, the Fast group showed enrichment in stress adaptation and biosynthesis pathways, including translation, ribosome biogenesis, homologous recombination, and AMPK signaling (Figure 7B).

At P3, the Zn group continued to show enrichment in metabolic pathways, particularly carbohydrate metabolism, and also exhibited differences in protein homeostasis pathways, such as protein folding, sorting, and degradation. The Fast group continued to exhibit enrichment in stress response and biosynthesis pathways established during P2 (Figure 7B).

### 3.8 Fast and high-zinc exhibit different serum metabolite profiles during the induced molting periods

**FIGURE 8:**
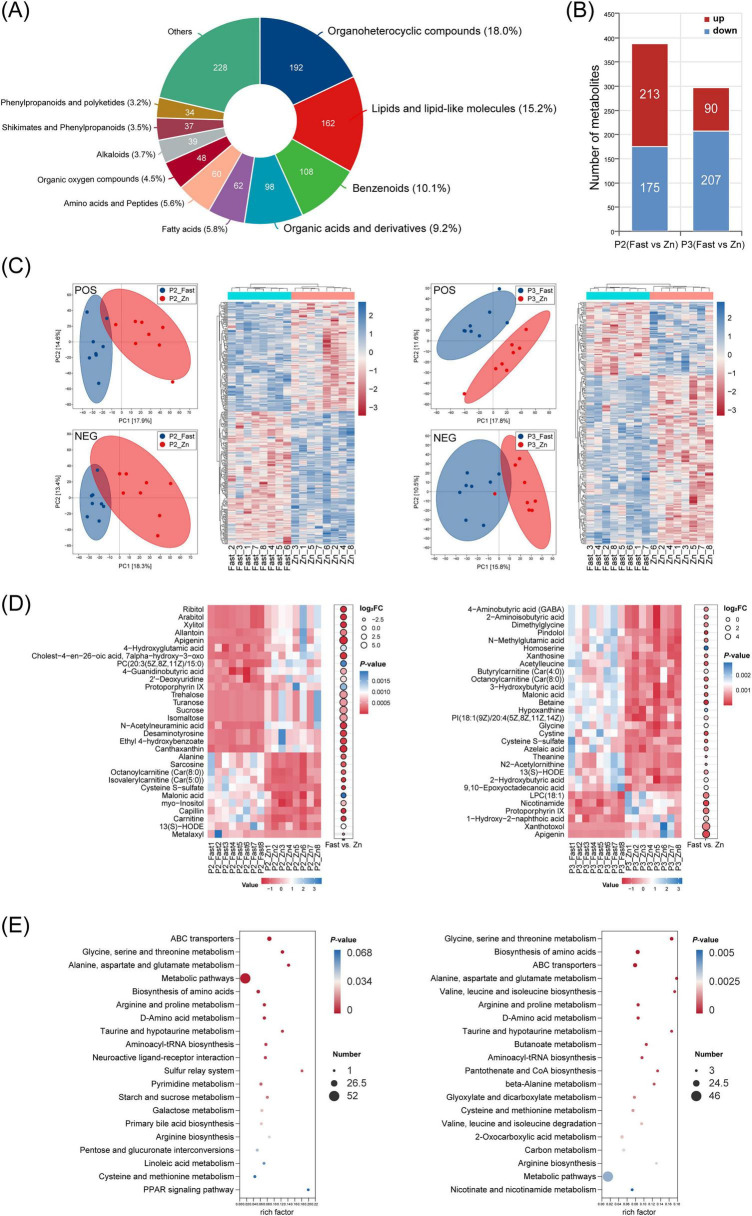
Serum metabolome overview, group separation, key features, and pathway enrichment. **(A)** Chemical-class composition of detected metabolites (percentage and counts). **(B)** Numbers of differential metabolites (up/down) for P2 (Fast vs. Zn) and P3 (Fast vs. Zn) using the criteria described in Methods. **(C)** PCA score plots (positive and negative) and corresponding heatmaps of all quantified features, illustrating separation between groups at P2 and P3. **(D)** Heatmaps of representative differential metabolites (top features) with log2 fold-change and adjusted *P-*values indicated. **(E)** KEGG pathway enrichment dot plots for differential metabolites at P2 (left) and P3 (right). Serum metabolomic analyses were performed using *n* = 8 biological replicates per treatment per stage.

Across all samples, organoheterocyclic compounds (18.0%), lipids and lipid-like molecules (15.2%), and benzenoids (10.1%) were the predominant chemical classes (Figure 8A). Unsupervised PCA and hierarchical clustering in both ionization modes showed clear separation between the Fast and Zn groups at P2 and P3 (Figure 8C), consistent with extensive treatment-associated differences in metabolite profiles (388 differential metabolites at P2 and 297 at P3; Figure 8B; Supplementary Table 2).

At the class level, treatment effects at P2–P3 were primarily reflected in amino acid–related metabolites and carbohydrate/organic-acid intermediates, with additional contributions from lipid-related features (Supplementary Table 2). Representative metabolites illustrating these shifts are shown in Figure 8D, while the complete lists (including fold changes and statistics) are provided in Supplementary Table 2.

KEGG over-representation analysis highlighted pathways shared across both stages, including ABC transporters; glycine, serine and threonine metabolism; alanine, aspartate and glutamate metabolism; global metabolic pathways; and amino-acid biosynthesis (Figure 8E). Collectively, these results suggest that zinc feeding preferentially supports amino-acid–centered metabolic remodeling of the serum metabolome during induced molting.

### 3.9 Integrated correlation analysis of microbiota, microbial functions, and serum metabolites during molting

**FIGURE 9:**
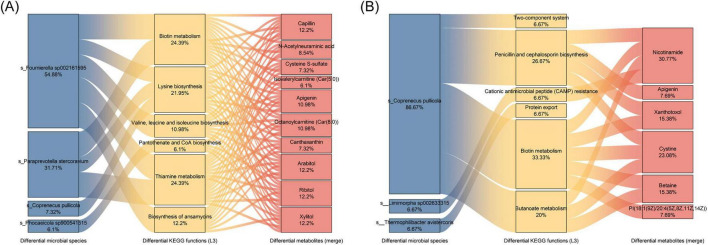
Integrated species-function-metabolite networks linking microbiota to host metabolism. Sankey diagrams connecting differential microbial species (left), differential KEGG functions (L3) (center), and differential serum metabolites (right) for P2 **(A)** and P3 **(B)** (Fast vs. Zn). The top 10 differential metabolites were selected based on VIP values (VIP > 2.0, *P* < 0.05) and possessing KEGG IDs; Pathway tracing between microbes, functions, and metabolites was performed based on the results from Spearman correlation analysis (| rho| ≥ 0.5, *P* < 0.05). Edge widths reflect relative contribution; percentages next to nodes indicate their proportion within the respective layer.

Within the Zn group, integrative Spearman correlation analysis (| rho| ≥ 0.5, *P* < 0.05) linked differentially abundant cecal taxa to KEGG L3 functions and serum metabolites at P2 and P3 (Figure 9).

P2. Enriched taxa, including *Fournierella sp002161595*, *Paraprevotella stercoravium*, *Coprenecus pullicola*, and *Phocaeicola sp900541515*, were positively associated with cofactor and amino-acid pathways such as biotin metabolism, lysine biosynthesis, and thiamine metabolism, as well as valine/leucine/isoleucine biosynthesis, pantothenate and CoA biosynthesis, and ansamycin biosynthesis. These functional nodes, in turn, correlated with metabolites including capillin, apigenin, octanoylcarnitine, arabitol, ribitol, and xylitol (Figure 9A).

P3. *Coprenecus pullicola* emerged as a central hub taxon, strongly associated with biotin and butanoate metabolism and penicillin/cephalosporin biosynthesis, alongside protein export, CAMP resistance, and two-component signaling. These functions were linked to metabolites such as nicotinamide, apigenin, xanthotoxol, cystine, and betaine (Figure 9B).

Collectively, these stage-resolved associations suggest coordinated microbiota–function–metabolite changes during molting induction under the high-zinc regimen.

## 4 Discussion

Previous studies suggested that a body weight loss of 25–30% was optimal for induced molting in laying hens, as any deviation from this range could hinder post-molt productivity. In this study, we used a 30% body weight reduction combined with complete cessation of egg production as a marker to define the molting period. This approach ensured better recovery and improved post-molt laying performance, consistent with previous findings (Tiwary et al., 2019; Onbaşılar et al., 2020).

Both high-zinc diets and fasting effectively induced molting, as evidenced by the cessation of egg production and its subsequent resumption upon refeeding. This was in line with Silva-Mendonça et al. (2015), who showed that both methods could trigger reproductive rest and rejuvenation. The enhanced post-molt productivity likely resulted from physiological reprograming during molting, where controlled weight loss and reduced adipose tissue allow for reproductive tract regression and rejuvenation. During refeeding, nutrient replenishment, especially proteins, lipids, and minerals, supports follicular development and yolk synthesis, restoring egg production (Airin et al., 2020).

Hormones such as E2, LH, TRH, IGF-1, and TGF-β are critical in assessing reproductive potential. Decreased estrogen levels in older layers can affect bone remodeling and calcium absorption, thereby affecting eggshell quality, while inducing molt usually increases estrogen levels and improves egg and eggshell quality (Ma et al., 2025). Both high-zinc diets and fasting treatments significantly improved post-molt egg quality, possibly by restoring circulating E2 levels. These restored levels stimulate calcium-binding proteins, improve intestinal calcium absorption, and increase eggshell strength (Goltzman et al., 2018). However, some deviations from previous reports were observed, including reductions in Haugh unit, albumen height, and yolk color. These changes likely reflect transient nutrient constraints and metabolic reallocation during the induction/recovery period, which may temporarily limit amino-acid substrates required for albumen protein synthesis (Jiang et al., 2019). Consistent with this interpretation, our metabolomics revealed enrichment of glycine/serine/threonine-related pathways under the high-zinc regimen during the induction stage (P2–P3), which likely reflects metabolic reprograming under the molting stimulus rather than increased substrate availability for albumen deposition. Notably, hens on the high-zinc regimen exhibited significantly higher E2 levels and increased LH concentrations during the recovery phase, consistent with earlier studies (Braw-Tal et al., 2004), although this pattern is broadly consistent with earlier studies, differences in the magnitude and timing of hormonal recovery across studies may reflect variation in hen age, induction protocol, lighting conditions, and the specific recovery stage at which samples were collected. This increase likely reflects ovarian tissue regeneration, supported by a transient rise in IGF-1, which promotes follicular renewal and stimulates E2 production (Waszkiewicz et al., 2020).

Zinc supplementation can also promote LH secretion and follicular development, further supporting reproductive recovery (Sandhu et al., 2008). TGF-β, which regulates tissue repair and apoptosis, exhibits different patterns with different treatments. High zinc levels may help attenuate ovarian functional decline and support follicular development. This effect may be related to the regulation of TGF-β-associated fibrotic remodeling, thereby potentially limiting excessive collagen deposition in reproductive tissues and contributing to better eggshell quality (Li et al., 2019; Liu et al., 2024). In contrast, fasting causes increased CORT levels, inhibits TRH secretion, and disrupts the function of the hypothalamic-pituitary-thyroid axis (Keshavarz and Quimby, 2002). However, the high-zinc group experienced a smoother negative energy balance, avoided significant increases in corticosterone levels, and supported more stable thyroid function.

Inflammatory markers such as IL-1β, IL-8, IL-6, CXCL1, LPS, and DAO are important for assessing immune and intestinal health. Overexpression of IL-1β interferes with follicle development and eggshell mineralization (Nii et al., 2018). In our study, IL-1β levels were significantly reduced in the Zn group, suggesting that the high-zinc regimen was associated with lower inflammatory stress and improved immune homeostasis, which is consistent with previous research (Akbari Moghaddam Kakhki et al., 2018). With respect to barrier-related indices, the effects of zinc were stage-dependent. Compared with fasting, zinc feeding was associated with lower LPS and DAO at several stages, indicating potential mitigation of endotoxin burden and epithelial injury; however, this pattern was not uniform across all time points (LPS was higher in Zn at P4). Collectively, these results suggest that zinc supplementation may help maintain intestinal integrity during induced molting, while the magnitude and direction of changes in circulating LPS/DAO can vary across stages.

During the molting process, the intestinal microbiota changed significantly, especially in the Zn group. During the P2 period, the high-zinc regimen promoted the diversity and functional enrichment of the microbiota, in which beneficial bacteria were dominant, such as *Coprenecus pullicola*, *Paraprevotella stercoravium*, and *Fournierella sp002161595*. These taxa have been linked to intestinal immune modulation and mucosal homeostasis in poultry or other hosts, suggesting that zinc may favor a microbial milieu conducive to stress adaptation and gut stability (Li et al., 2022; Liu et al., 2023; Clausen et al., 2024). At P3, the beneficial bacteria in the Zn group were enriched earlier, while the microorganisms in the fasting group recovered later. This may contribute to the faster physiological recovery and better production performance observed in the high-zinc group.

In terms of metabolic pathways, the high-zinc regimen enriched amino-acid metabolism and cofactor biosynthesis, supporting nutrient retention and tissue repair during molting. Zinc also serves as a cofactor for antioxidant enzymes, enhancing oxidative defense and limiting tissue damage (Liu et al., 2015). By contrast, fasting activated cellular stress–adaptation pathways, including AMPK signaling, thereby promoting catabolic processes such as hepatic fatty-acid oxidation and ketogenesis (Ruppert and Kersten, 2024; Storoschuk et al., 2024). Serum metabolomics further indicated a central role for amino-acid metabolism during molting, with enrichment of metabolites such as glycine, serine, and glutamate that support one-carbon metabolism and protein turnover required for feather regeneration and cellular proliferation (Li and Ye, 2020; Pan et al., 2020). Together, these shifts suggest a coordinated adaptation that preserves essential functions while reprograming metabolism to meet biosynthetic demands under transient nutrient constraint.

Spearman correlation analysis within the high-zinc group revealed strong links between bacterial taxa, metabolic pathways, and serum metabolites. For example, *Fournierella sp002161595* and *Paraprevotella stercoravium* positively correlated with biotin and lysine biosynthesis, supporting microbial contributions to amino acid and cofactor metabolism. *Coprenecus pullicola* emerged as a central metabolic hub, coordinating pathways related to redox balance and cellular repair, further supporting a potential association between the high-zinc associated microbiota and physiological stability during molting.

However, several limitations should be acknowledged. First, the present study employed a classical 2% ZnO molting regimen to compare two established molting models and their associated microbial, metabolic, and physiological responses, rather than to perform a zinc dose-response or threshold evaluation. Therefore, under the present design, the current data do not allow determination of the minimum effective dose or action threshold of ZnO. In addition, it remains unclear how long the post-molt production peak associated with the high-zinc regimen can be sustained under practical conditions, which warrants extended real-world monitoring. Future studies should also identify the ovarian targets and signaling pathways associated with the high-zinc molting response, and incorporate graded ZnO levels to better define the effective dose range and optimize nutritional strategies for molt induction.

## 5 Conclusion

In conclusion, our findings suggest that the high-zinc regimen (2% ZnO) promote a beneficial gut microbiota and enhance metabolic pathways that support immune function, nutrient metabolism, and reproductive recovery. This strategy offers a more welfare-friendly alternative to traditional feed withdrawal for inducing molting in commercial laying hens, facilitating faster recovery and improved post-molt productivity.

## Data Availability

The raw sequencing data are available in the NCBI SRA (https://www.ncbi.nlm.nih.gov/sra) under accession number PRJNA1240360.
